# Job Stress and Burnout Among Employees Working in Terrorist-Ridden Areas

**DOI:** 10.3389/fpsyg.2021.667488

**Published:** 2021-07-01

**Authors:** Shuaib Ahmed Soomro, Akhtiar Ali Gadehi, Xu Hongyi Xu, Sarfaraz Ahmed Shaikh

**Affiliations:** ^1^Department of Business Administration, Sukkur Institute of Business Administration (IBA), Sukkur, Pakistan; ^2^Bahria University Karachi Campus, Karachi, Pakistan; ^3^School of Management, Wuhan University of Technology, Wuhan, China; ^4^Indus Center for Sustainable Development, Karachi, Pakistan

**Keywords:** job stress and strain, organizational injustice, burnout - professional, psychology, terrorism salience, effort reward imbalance model

## Abstract

This article examines the relationship of effort-reward imbalance (ERI) on employee stress by considering job burnout (BO), organizational (in)justice (OJ), and sensitivity to terrorism (STT). This study uses the effort-reward imbalance (ERI) framework as a job stress model. After describing terrorism and a brief discussion on organizational justice and some of its challenges, we introduced “sensitivity to terrorism” as a moderator in the ERI framework. Using a 432 sample size of questionnaire data collected from two big cities where terrorist attacks are rampant and received during a period when terrorist attacks were at a peak. After analyzing data in EFA, results from the hierarchical regression analysis provided support for our developed model. Overall, the statistical model is significant (*p* < 0.05). We found significant relationships between ERI and facets of BO. Organizational (in)justice mediated the influence of ERI on facets of BO. We also found that STT significantly moderated ERI and facets of burnout. The article concludes with some implications and guidelines for future research.

## Introduction

Stress diminishes the ability to perform because of its adverse effect on health (Fischer et al., 2006; Marmot, 2015; Harlow and Lawler, 2018). Stress in the workplace emerges from external sources such as changes in political setups (Labrague et al., 2017), advancement in technology (Bliese et al., 2017; Carnall, 2018), changes in the economy (De Jonge and Dormann, 2017); and from within the organization due to lack of resources, organizational justice, gender, and ethnic bias.

Current work focuses on the internal and external issues that ignite stress levels with a specific focus on organizational justice, effort-reward imbalance, and acts of terrorism at or near the workplace (Czinkota et al., 2010). The act of terrorism complicates and slows down economic activities since it makes challenging for a workplace to function smoothly. Engaging employees in terrorism-ridden areas is a big challenge since terrorism adversely affects job commitment, produces turnover intentions, lowers performance, and ultimately ends in health problems (Hobfoll et al., 2007).

Incidents of terrorism foster uncertainty that affects employees and is considered one of the primary determinants of stress (Brandon and Silke, 2007). Uncertain factors are believed to be the fundamental determinant of job stress. Moreover, employment in terrorism-ridden areas is often physically and emotionally demanding when one compares the priceless value of life with rewards, such as compensation, fringe benefits, job security, personal accomplishment, and overall satisfaction, which lead to job stress. Increased direct and indirect incidents near and around workplaces have created work-life complications, affecting the performance of the organization as well as employee well-being (Canetti-Nisim et al., 2009; Malik et al., 2014).

The relationship between employee job stress working in terrorism-ridden areas is less explored (Bader et al., 2015). The extant literature on the effects of terrorism on employee behavior and the working practices of organizations is limited and empirical evidence is relatively low in the context of Pakistan (Soomro et al., 2020). The present study attempts to address this gap by studying Pakistan's businesses that are affected by terrorist incidents. This study uses the ERI framework and takes a focused view of incidents of discontinuous terrorism incidents around the workplace.

The aim of the study is first to describe the ERI framework, which provides a valuable approach to studying working environments in terrorism-ridden areas. Second, we suggest that organizational justice, characterized as the organization's behavior and the employee's resulting behavior, for example, employers making workers redundant, an employee may feel a sense of injustice with a consequent change in output and may increase burnout. We propose that a fair process of organizational justice will make employees more prone to work and experience less burnout at work.

## ERI and Job Burnout

Siegrist (2016) developed the ERI model to identify the health-adverse effects of stressful psychosocial work and employment conditions in developed and rapidly developing countries. The word “effort” is used in its broadest sense to refer to all efforts the employee made, while the term “reward” is used to describe all rewards received in exchange for efforts. The imbalance between high effort and low rewards received is likely to have negative well-being, which increases stress (Siegrist et al., 2004; Lange et al., 2005; Van Vegchel et al., 2005; De Jonge et al., 2008). A balance between effort spent and reward received is likely to promote well-being and high health (Montano et al., 2016). The disequilibrium may cause a condition of emotional distress, which can prompt strain responses.

In an organizational context, Maslach and Jackson (1981) described burnout as an outcome of job stress. Long-lasting stress at work is often due to prolonged involvement and association with unfavorable and emotionally worrying working conditions (Ahola et al., 2009; Klein et al., 2010). It results from excessive demands at work, such as mental distress, sleeplessness, anger, rumination, and so forth (Leiter and Maslach, 2008; Yao et al., 2015).

The current study takes burnout as a three-dimensional variable, namely: emotional exhaustion, which refers to the feeling of physical or mental tiredness at work (Maslach and Leiter, 2017); depersonalization, referring to those who show impersonal reactions to their colleagues, and reduced accomplishment; refers to those who feel that they have little or no feeling about their achievements (Schaufeli et al., 2017).

The mismatch in effort-reward ratio ends in burnout (Leiter and Maslach, 2008), increased absenteeism, and lower performance (Baruch-Feldman et al., 2002). A loss of feelings about co-workers increases tiredness, and dissatisfaction toward growth at work are some of the indicators of burnout of an employee at work (Schaufeli and Greenglass, 2001; Schaufeli and Bakker, 2004; Leiter and Maslach, 2008).

The ERI framework with burnout provides a valuable approach to study toxic elements (terrorism) in the work environment. It suggests that high effort and low reward imbalance may have enduring effects, causing a gradual depletion of individuals' emotional resources and well-being (Shirom, 1989; Peter et al., 1998; Kivimäki and Siegrist, 2016). Therefore, the study proposes the following hypothesis based on facets of burnout.

*Hypothesis 1(a); High efforts and low rewards in work environments in terrorism-ridden areas will be significantly associated with emotional exhaustion*.
*Hypothesis 1(b); High efforts and low rewards in work environments in terrorism-ridden areas will be significantly associated with depersonalization*.
*Hypothesis 1(c); High efforts and low rewards in work environments in terrorism-ridden areas will be significantly associated with reduced accomplishment*.

## Role of Organizational Justice as a Mediator

Organizational justice refers to employees' perceptions of fairness in the workplace (Greenberg, 1990). It represents an employee's desire to obtain, establish or indicate rules that are equally applicable throughout the organization. Uniform rules are valuable indicators of the fairness of procedural outcomes, interpersonal treatment, and more contemporary perspectives cast a broader net. Prior literature on the organization justice construct has focused on the degree to which employees view themselves as fairly treated, more recent theories consider employees' reactions to the treatment of others (Elovainio et al., 2005b; Ambrose and Schminke, 2009). According to the ERI conceptual framework, more research is required on the justice construct because it causes adverse effects due to the developing countries' employment conditions.

Moreover, the relationship between ERI and burnout is mediated by organizational justice (Elovainio et al., 2005a). The mismatch in efforts and reward fosters a sense of injustice. In the long run, the sense of injustice opens up room for rumination and leaves the employees in a state of neglect. Neglection is a source of depersonalization and emotional exhaustion. Unbalanced exchanges in contexts between efforts and rewards make the work more unpredictable. It emphasizes the adequacy of the effort-reward ratio (Van den Bos and Lind, 2002; Elovainio et al., 2005a). When environmental uncertainty increases and effort-reward imbalance is high, employees are more likely to have lower well-being and symptoms of burnout. Hence, when organizational injustice stays, and terrorist incidents are at a high, one's desire is to keep alive with all one's resources. The following hypothesis are proposed based on the above context.

*H 2(a): Organizational justice will significantly mediate the relationship between effort-*
*reward imbalance and emotional exhaustion relationship*.
*H 2(b): Organizational justice will significantly mediate the relationship between effort-*
*reward and depersonalization relationship*
*H 2(c): Organizational justice will significantly mediate the relationship between effort-*
*reward and reduced accomplishment relationship*.

## Role of Sensitivity to Terrorism (STT) as a Moderator

Fear in the organizational context creates a similar impact on the personality of employees. However, not all employees are the same personality traits. Terror management theory suggests that organizations may decide to have comfort and avoid employees' thoughts of death. Terror management theory (TMT) posits that in general, incidents of terrorism cause stress and death related consequences, in particular, have mixed feelings of vulnerability (Greenberg et al., 1986). However, the degree of vulnerability may not remain the same for all employees. Some individuals may tend to be more sensitive and easily affected by a situation, while others may show a less sensitive reaction toward it (Lazarus, 1995). The indirect effects of terrorism, such as emotional harm, depersonalization, and reduced accomplishment, are widespread, damaging the well-being of employees (Burke and Mikkelsen, 2006; Schaufeli et al., 2017).

STT moderates the relationship between organizational injustice and facets of burnout. In the sense that higher benefits should be associated with lower job burnout in the study context. The reason behind this is when employees are in trouble, then they expect a reasonable effort-reward ratio from the employers. They also hope employers support them and provide guarded security and cognitive therapy when threats are outward. Therefore, in this study, STT is predicted to moderate the direct relationship between ERI–burnout and organizational injustice-burnout relationships. Thus, it is proposed:

*Hypothesis 3(a); Sensitivity to terrorism will moderate the relationship between effort-reward imbalance and emotional exhaustion*.
*Hypothesis 3(b); Sensitivity to terrorism will moderate the relationship between effort-reward imbalance ratio and depersonalization*.
*Hypothesis 3(c); Sensitivity to terrorism will moderate the relationship between effort-reward imbalance ratio and reduced accomplishment*.
*Hypothesis 4(a); Sensitivity to terrorism will moderate the relationship between organizational injustice and emotional exhaustion*.
*Hypothesis 4(b); Sensitivity to terrorism will moderate the relationship between the organizational injustice ratio and depersonalization*.
*Hypothesis 4(c); Sensitivity to terrorism will moderate the relationship between organizational injustice and reduced accomplishment*.

## Methodology—Research Context and Data Collection

The study aims to measure the effects of predictors and comprises data from terrorist-ridden areas. For instance, earlier research on terrorist incidents, in general, provides surface descriptions of the problem. Prior research on this subject is based on a single incident, its consequences focus on a western context (Ryan et al., 2003, p. 11). Besides, previous research on terrorism is relatively based in a stable environment, i.e., USA. Hence, in this study, through focused investigations, we analyzed the influence of terrorism and how it is affecting employees at work in the context of developing countries.

Two cities Quetta and Peshawar, are targeted for data collection since these cities sustained multiple terrorist incidents. The educational institutes make up the target population, whereas the purposive sampling method is used for data collection. We used purposive sampling to undertake an in-depth analysis of the conceptual framework. The academic staff took part from the two sample cities (Peshawar and Quetta) following the Army Public School (APS) attack (December 2014). Sample respondents who were affected by the attack, directly and indirectly, form the study respondents. This helped to collect data from the organizations and employees to fulfill the study's aims.

A total of 950 survey questionnaires in the English language were distributed. At the start of data collection, people received a cover letter in the English language. This is the official language of the country and researchers use it for the purposes of studies. All the participants were assured of confidentiality and anonymity, and provided consent before their participation. Each participant completed a survey with items related to the effort-reward imbalance, organizational justice, sensitivity to terrorism, emotional exhaustion, depersonalization, and lack of personal accomplishment. The questionnaire included demographics (such as sex, age, education, and employment period) and questions on study variables. The response rate was 45 percent (i.e., 432/950).

## Scales and Measures

### Effort-Reward Imbalance

Overall, 23 scale items were used to measure the effort-reward framework. This scale deals with three major components of ERI: effort, reward, and over-commitment (Siegrist et al., 1997). A six item scale measured the effort component, 11 questions measured the reward component. It was further split into three parts, namely; monetary fulfillment, status control, and esteem reward. In this research, we have used the effort and reward components of the ERI framework. All scales measured in this research used a five-point Likert scale.

### Organizational Justice

A 6-item questionnaire measured the perceived overall justice (POJ) based on the work of (Lind, 2001) and (Colquitt and Shaw, 2005). The respondents answered the questionnaire by measuring the POJ scale to assess the personal justice experience and general fairness of the organization. The three items were evaluated as employees' personal justice experiences; “Overall, I'm treated fairly by my organization;” “In general, I can count on this organization to be fair;” “In general, the treatment I receive around here is fair.” The remaining three items assessed the fairness of the organization generally; “Usually, the way things work in this organization are not fair;” “For the most part, this organization treat its employees fairly;” “Most of the people who work here would say they are often treated unfairly.”

### Sensitivity to Terrorism

A 3-item scale measured STT to terrorism based on the work of Reade (2009). These items include “I seem to lose enthusiasm for work whenever I get news of terrorist incident,” “I feel tenser at work when there is a fresh incident,” and “I sometimes miss work or find it difficult to perform my job well due to the mood created by the terrorist incident.”

### Maslach Burnout Inventory (MBI)

The inventory questionnaire was developed by Maslach and Jackson (1981). It measured the three burnout components, i.e., emotional exhaustion (e.g., “I feel like I am at the end of my rope”), depersonalization (e.g., “I've become more callous toward co-workers' lives through my work”), and reduced accomplishment. Overall, a 22 item scale measured three facets of burnout inventory.

### Demographic Variables

Respondents were asked to mention sex, age, education, and work experiences. The sex is categorized as (1) Male and (2) Female. Age groups were computed on the mean score using categories of (1) <25 years, (2) 26–34 years, (3) 35–44 years, (4) 45-54 years, (5) 55, and above. Education categories as (1) College-level, (2) Bachelor's, (3) Masters, and (4) M.Phil./M.S. Finally, experience is categorized as (1) 1–5 years, (2) 6–10 years, (3) 11–15 years, (4) 16–20 years, (5) 21>. We controlled for sex, age, education, and experience as these might influence ERI and burnout relationship.

In Table 1, the results stand for mean, std. Deviations, intercorrelations, and the reliabilities on the diagonal. Out of 432 questionnaires, 247 are reported male (57%); 185 (43%) are female, 138 (32%) are reported between 35 and 44 years. Furthermore, 138 (32%) of the sample had working experience of between 5 and 10 years, and 355 (82%) of the sample comprised a master's degree.

**Table 1:** Mean, standard deviations, correlations, and reliabilities.

	**Mean**	**S.D**	**1**	**2**	**3**	**4**	**5**	**6**	**7**	**8**	**9**	**10**	**11**
1. Sex	1.33	0.47	1.00										
2. Age	2.72	1.13	−0.205^**^	1.00									
3. Education	2.22	0.49	0.02	−0.096^*^	1.00								
4. Experience	2.37	1.14	−0.08	0.098^*^	0.08	1.00							
5. Effort	3.29	0.78	0.097^*^	−0.157^**^	0.109^*^	0.118^*^	0.81						
6. Reward	3.41	0.77	0.08	−0.185^**^	0.107^*^	0.09	0.434^**^	0.92					
7. OJ	3.27	1.16	0.125^**^	−0.202^**^	0.135^**^	0.252^**^	0.393^**^	0.322^**^	0.93				
8. STT	3.46	0.87	0.06	−0.102^*^	0.103^*^	0.184^**^	0.252^**^	0.280^**^	0.354^**^	0.79			
9. EE	2.51	0.95	0.01	−0.06	0.05	0.04	0.125^**^	0.08	0.404^**^	0.09	0.92		
10. DP	2.59	0.96	−0.01	−0.06	−0.01	0.00	0.09	0.111^*^	0.333^**^	−0.01	0.403^**^	0.91	
11. PA	2.56	1.13	−0.02	−0.06	−0.01	0.102^*^	0.09	0.08	0.495^**^	0.04	0.297^**^	0.322^**^	0.96

*Figures on main diagonal are Cronbach's alpha reliabilities. The mean and Std. Deviation were recorded. Sex: male = 1, female = 2; Age: 1 = <25, 2 = 26–35, 3 = 36–45, 4 = over 46–55, 5 = 56 and above; Experience: 1 = 1–5 years, 2 = 6–10 years, 3 = 11–15 years, 4 = 16–20 years, 5 = 21>; Education: 1 = College level, 2 = Bachelor's, 3 = Masters, 4 = M.Phil. and above*.

** *p < 0.01 level*.

* *p < 0.05 level*.

### Analysis and Results

All variables examined before the primary analysis—the normality of the entire data and detection of outliers. To analyze the direct, indirect, and total effects, we used the Process macro version described in Preacher and Hayes (2004). The procedures separately test the moderation and mediation hypotheses with 5,000 bootstraps used in the case of the mediator variable.

### Factor Analysis

Before testing the hypotheses, we used factor analysis to confirm the adequacy of selected variables in this context. The value of the KMO is.91 and Bartlett's test of sphericity is significant (*p* < 0.001). The analysis extracted seven factors based on the eigenvalue greater than 1 accounting for 67.84%. Factor 1 formed of eight items explained 25.13% of the variance with loadings from 0.825 to 0.906. Factor 2 consists of 11 items that explain 14.88% of the variance with loadings from 0.644 to 0.822. Factor 3 comprised of seven items that explained 8.70% of the variance with loadings from 0.716 to 0.858. Factor 4 consists of seven items that explained 6.74% of the variance with loadings from 0.602 to 0.863. Factor 5 comprises six items that explained 5.3% per cent of the variance with a factor from 0.750 to 0.868. Factor 6 consists of six items that explain 4.16% per cent of the variance with loadings from 0.362 to 0.890. Factor 7 consists of three items that account for 2.98% of the variance with loadings from 0.734 to 0.775. The whole analysis found seven clear patterns, and all are independent of one another (please see Table 2).

**Table 2:** Pattern matrix.

**Items**	**Personal acquisition**	**Reward**	**Emotional exhaustion**	**Depersonalization**	**Org. Justice**	**Effort**	**Situation to terror**
EF1						0.362	
EF2						0.755	
EF3						0.89	
EF4						0.792	
EF5						0.429	
EF6						0.454	
RW1		0.692					
RW2		0.774					
RW3		0.789					
RW4		0.739					
RW5		0.75					
RW6		0.75					
RW7		0.644					
RW8		0.67					
RW9		0.79					
RW10		0.669					
RW11		0.822					
OJ1					0.833		
OJ2					0.849		
OJ3					0.868		
OJ4					0.785		
OJ5					0.75		
OJ6					0.821		
ST1							0.734
ST2							0.775
ST3							0.738
ME1			0.755				
ME2			0.818				
ME3			0.835				
ME4			0.757				
ME5			0.716				
ME6			0.84				
ME7			0.858				
MD1				0.849			
MD2				0.863			
MD3				0.829			
MD4				0.602			
MD5				0.731			
MD6				0.843			
MD7				0.638			
MP1	0.895						
MP2	0.847						
MP3	0.847						
MP4	0.843						
MP5	0.825						
MP6	0.887						
MP7	0.906						
MP8	0.876						

### Hypotheses Testing

#### Direct Effects

The relationship between ERI ratio and emotional exhaustion (EE), Depersonalization (DP), and reduced accomplishment (PA) were tested separately. All relationships are significant with *B* = 0.15, *P* < 0.00 for ERI and EE; *B* = 0.11, *P* < 0.06 for ERI and DP; and *B* = 0.13, *P* < 0.05 for ERI and PA. The relationship between ERI and organizational justice (OJ) stands significant at *B* = 0.59 and *P* < 0.005. The path from OJ to all dimensions of stress such as EE, DP, and PA was tested. In general, all direct hypotheses after investigation were found to be significant between OJ and EE (*B* = 0.33; *p* < 0.05^**^), between OJ and DP (*B* = 0.27; *p* <0.05^**^), between OJ and PA (*B* = 0.48; *p* < 0.05^**^) (see Table 3).

**Table 3:** Summary of direct effects.

	**OJ**				**EE**				**DP**				**PA**			
	***B***	***T***	***P***	***R^**2**^***	***B***	***T***	***P***	***R^**2**^***	***B***	***t***	***P***	***R^**2**^***	***B***	***t***	***P***	***R^**2**^***
ERI	0.59	8.85	0.00^**^	0.16	0.15	2.61	0.00^**^	0.16	0.11	1.83	0.06^*^	0.08	0.13	1.84	0.05^*^	0.09
OJ					0.33	9.16	0.00^**^	0.17	0.27	7.32	0.00^**^	0.11	0.48	11.80	0.00^**^	0.24

** *p < 0.01 level*.

* *p < 0.05 level*.

#### Indirect Effects

The data supported hypothesis 2(a) regarding OJ's mediating effect between the ERI ratio and EE. The indirect effect of the ERI ratio, *via* OJ, was significant on EE (*B* = 0.589; *p* < 0.001). The mediation results used Preacher and Hayes (2004) bootstrapping method and confirmed that OJ mediates the relationship between ERI ratio and EE.

The data supported hypothesis 2(b) regarding OJ's mediating effect between ERI ratio and DP. The indirect effect of ERI ratio, *via* OJ was significant on DP (*B* = 0.561; *p* < 0.001). The data supported hypothesis 2(c) regarding the mediating effect of organizational justice (OJ) between the ERI ratio and PA. The indirect effect of ERI, *via* OJ was significant on EE (beta = 0.591; *p* < 0.001) (see Table 4).

**Table 4:** Summary of indirect effects.

	***B***	***T***	***P***	***R^**2**^***
ERI → OJ → EE	0.589	8.85	0.00^***^	0.16
ERI → OJ → DP	0.561	8.65	0.00^**^	0.15
ERI → OJ → PA	0.591	8.95	0.00^**^	0.17

** *p < 0.01 level*.

* *p < 0.05 level*.

*** *p < 0.001 level*.

#### Interaction Effects

**Figure 1:**
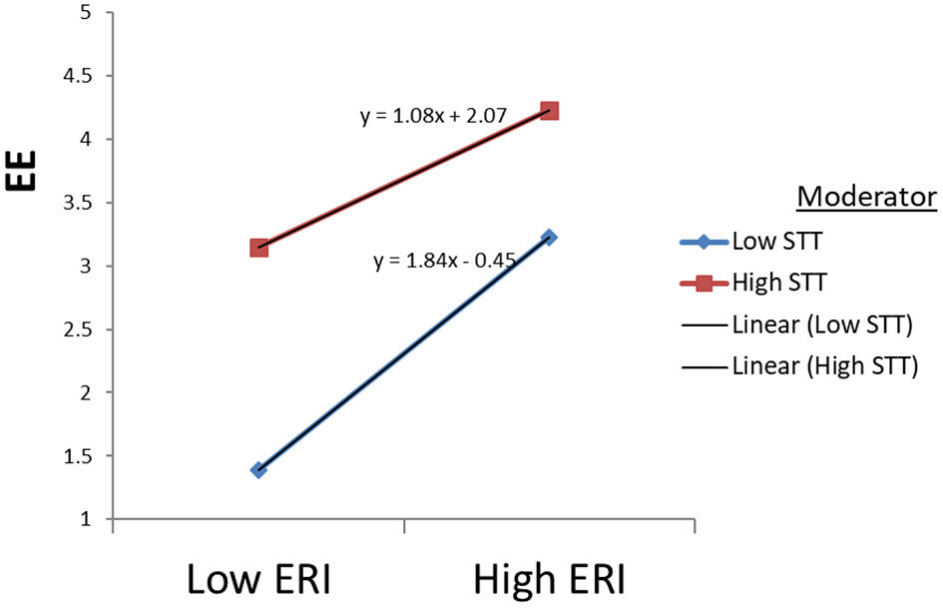
Moderating effects of STT on EE through ERI.

The data supported Hypothesis 3(a) about the moderating effect of the STT on the direct relationship between ERI ratio and EE. To rectify multicollinearity, we centered each of the two predictor variables, ERI and STT, by subtracting the sample mean from each variable before generating the interaction term. The interaction term was formed by multiplying the two-centered predictor variables: ERI^*^STT. The interaction term supported and showed a significant effect (*B* = −0.189; *p* < 0.001). To establish the direction of the supported moderating effect of STT, we probed the significant interaction effect in a graph (see Figure 1). Overall, STT slightly dampens the negative relationship between ERI and EE in the graph.

**Figure 2:**
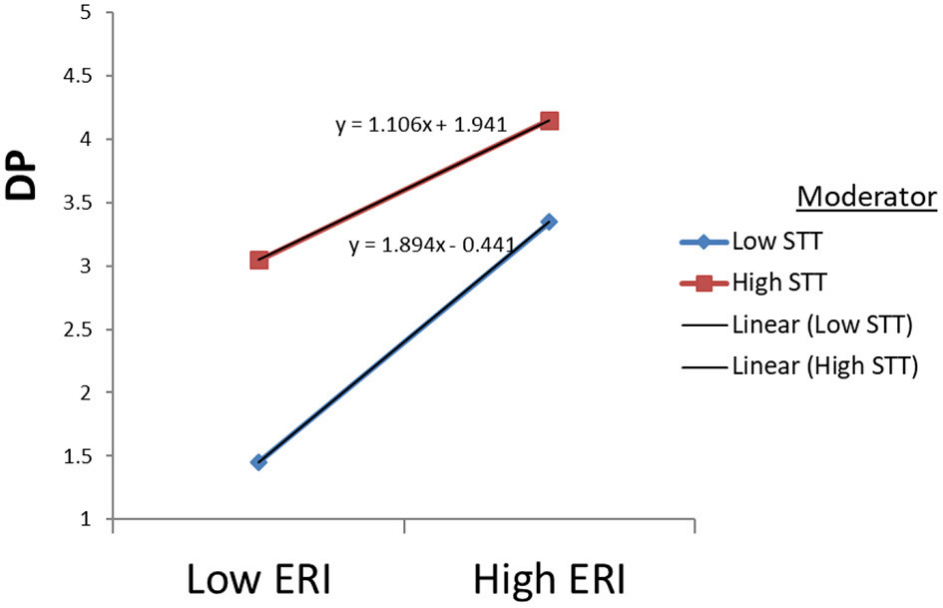
Moderating effects of STT on DP through ERI.

The data supported Hypothesis 3(b) about the moderating effect of the STT on the direct relationship between ERI ratio and DP. The interaction term was formed by multiplying the two-centered predictor variables: ERI^*^STT. The data showed a significant effect (*B* = −0.196; *p* < 0.001). Moreover, to set up the direction of the supported moderating effect of STT, we probed the significant interaction effect in a graph (see Figure 2). Overall, STT mildly dampens the negative relationship between ERI and DP in the graph.

**Figure 3:**
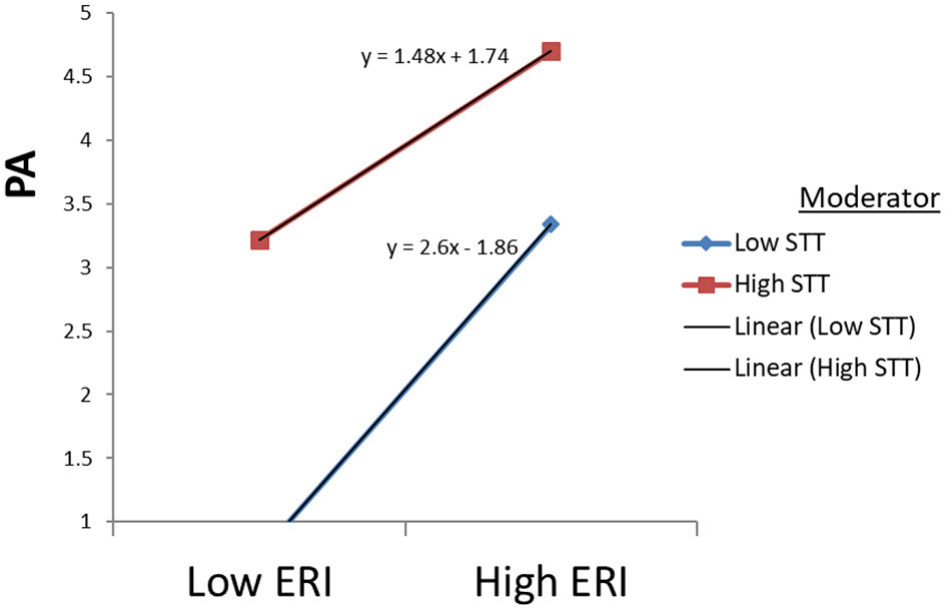
Moderating effects of STT on PA through PA.

The data supported Hypothesis 3(c) regarding the moderating effect of the STT on the direct relationship between ERI ratio and PA. The interaction term was formed by multiplying the two-centered predictor variables: ERI^*^STT. The data showed a significant effect (*B* = −0.284; *p* < 0.001). To set up the direction of the supported moderating effect of STT, we probed the significant interaction effect in a graph (see Figure 3). Overall, STT mildly dampens the negative relationship between ERI and PA in the graph.

Hypothesis 4(a) about the moderating effect of the STT on the direct relationship between OJ and EE was not supported. Therefore, it is not reported here.

**Figure 4:**
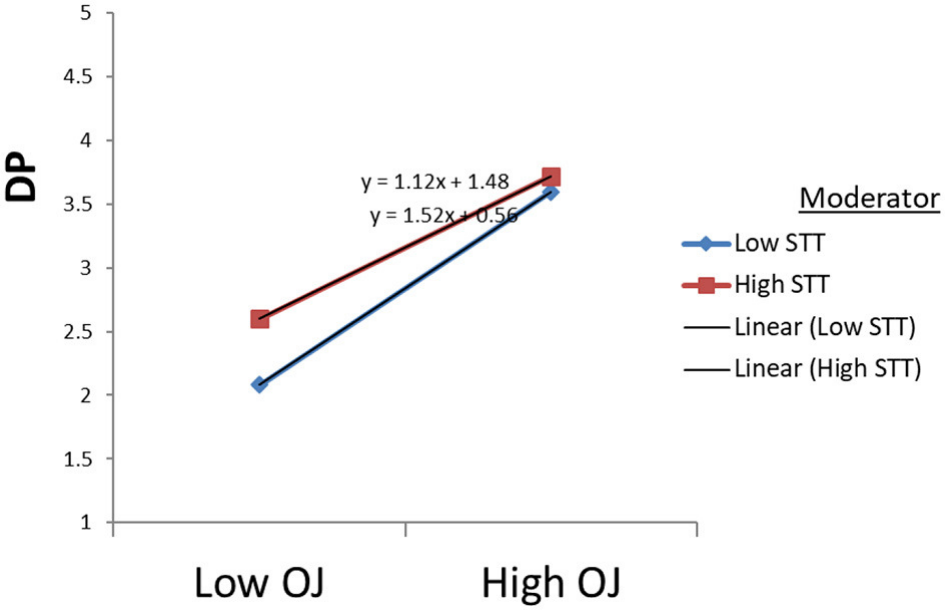
Moderating effects of STT on DP through OJ.

Hypothesis 4(b) regarding the moderating effect of the STT on the direct relationship between OJ and DP was supported. The interaction term was formed by multiplying the two centered predictor variables: OJ^*^STT. The data showed a significant effect (*B* = −0.107; *p* < 0.01). To establish the direction, we probed the significant interaction effect (see Figure 4). Overall, STT somewhat dampens the negative relationship between OJ and DP in the graph.

**Figure 5:**
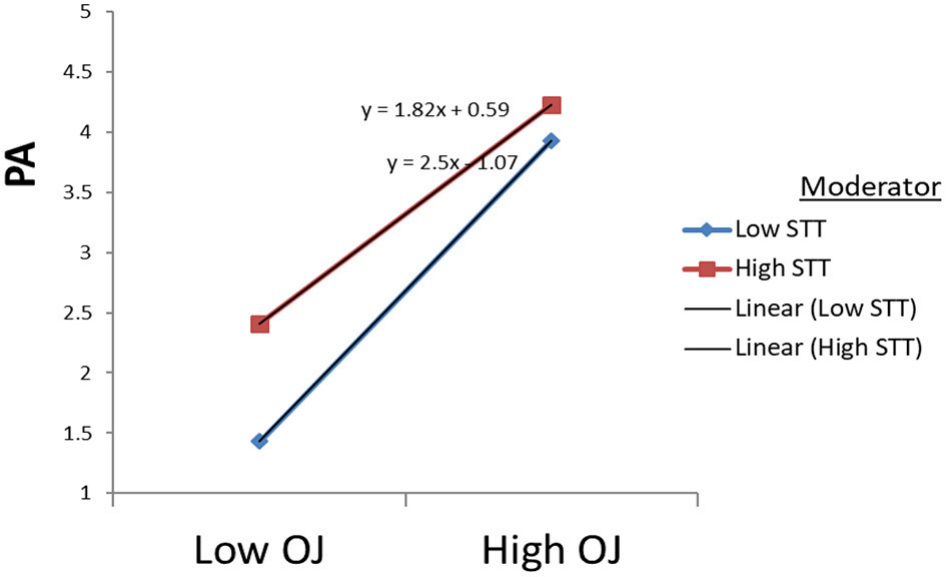
Moderating effects of STT on PA through OJ.

The data supported Hypothesis 4(c) about the moderating effect of the STT on the direct relationship between OJ and PA. The interaction term was formed by multiplying the two centered predictor variables: OJ^*^STT. The data showed a significant effect (*B* = −0.17; *p* < 0.001). To establish the direction, we probed the significant interaction effect (see Figure 5). Overall, STT slightly dampens the negative relationship between OJ and DP in the graph. A summary of the moderating effects is presented in Table 5.

**Table 5:** Summary of moderating effects.

	***B***	***P***
ERI*STT*EE	−0.18	0.00^*^
ERI*STT*DP	−0.19	0.00^**^
ERI*STT*PA	−0.28	0.00^*^
OJ*STT*DP	−0.10	0.00^*^
OJ*STT*PA	−0.15	0.00^**^

** *p < 0.01 level*.

* *p < 0.05 level*.

## Discussion and Conclusion

This study formulated a framework of suggestions for future directions in research (Bader et al., 2015; Soomro et al., 2018) and has successfully tested the ERI framework (Siegrist, 2002) to examine its impact on burnout. Three facets of burnout, including emotional exhaustion, depersonalization, and reduced personal acquisition were tested separately (Maslach and Jackson, 1981; Maslach, 2017). The motives surrounding all variables, including outcome, are thoroughly explained in the literature review. The results provided significant support for the proposed hypothesis in the study context, within the proposed theoretical framework. The framework examines the relationship between effort-reward imbalance, resultant job stress, and its impact on three facets of job burnout in terrorist-ridden areas. In the findings section, we contribute to literature on burnout by linking the crucial yet under-researched construct of the effort-reward imbalance for employees working in terrorist-ridden areas. This is one of the few studies that investigated the ERI framework using organizational justice as a mediator, STT as a moderator, and employee exhaustion, depersonalization, and reduced accomplishment as an output variable. Bader and Berg (2013) suggest the relevance of considering the STT variable for risky contexts.

Confirmation of hypothesis 1 about the relationship between ERI and emotional exhaustion, depersonalization, and reduced accomplishment in the study supports the argument that high effort and low rewards ratios lead to increased burnout among employees. The effort-reward imbalance framework suggests that failed reciprocity, in terms of high cost and low gain, increases job burnout, which has adverse effects on well-being (Siegrist et al., 1997). In existing research, employee higher efforts at the workplace cause their health as they receive no appropriate rewards in return. Under those circumstances, the feeling of physical danger and mental tiredness is pervasive in dangerous areas (Bader and Manke, 2018; Soomro et al., 2020). According to the literature, Bader and Berg (2014) also reported a significant negative relationship between sensitivity to terrorism, with compensation for expatriates (−0.113^*^). In this research, high efforts in the workplace in this context cause significant risk to employees' health and well-being. Moreover, Figures 1–5 describe the considerable effect of STT, which is weak for low sensitive employees and strong for high STT employees. Hence, the higher the sensitivity to terrorism, the higher the effect. It straightens the research on employee sensitivity working in terrorist-ridden areas.

The results support the hypotheses of the indirect effects of ERI on emotional exhaustion, cynicism, and reduced accomplishment through organizational justice. According to the ERI framework, uncertainty in terrorism-ridden areas creates a disturbance that makes it volatile for employees when working. Hence for employees looking for fair organizational policies, failure to do this may have adverse effects. Table 4 describes significant indirect effects. It is essential to understand that ERI wire organization justice is related to three facets of employee burnout. Organizational justice is a critical variable of the equation and is an appropriate way to test employee burnout in terrorism-ridden areas.

An uncertain environment underlies this indirect relationship in the context of this study. Firstly, uncertainty may be associated with the increased vulnerability of employees who attend their offices daily. Secondly, this uncertainty is sometimes resorting more frequently to determine the rewards received' adequacy, considering working in these contexts. Finally, the situation of risk as it is simultaneously uncontrollable could trigger attributive processes that would lead to saying the unfair organizational justice for the mismatch of effort-reward imbalance, which further affects the facets of employees' job burnout.

The data also support hypothesis 3, on the moderating effect of STT on the relationship between ERI ratio and three facets of job burnout. Thus accumulating evidence for the moderating hypothesis of the ERI framework, as also described by prior studies (Hämmig et al., 2012; Montano et al., 2016). In this study, results showed the weaker negative value of the interaction, which supports the idea that employees with higher sensitivity to terrorism show more signs of emotional exhaustion, depersonalization, and reduced accomplishment than employees with low sensitivity to terrorism, who show fewer effects. Overall, STT strengthens the relationship between the ERI ratio and facets of burnout.

Terrorism has received greater attention in business and management research after 9/11 (Howie, 2007; Soomro, 2019). It has a significant impact on many life characteristics, including employees' attitudes and behavior when working in high risk areas. Mainly, people living in ongoing terrorist-ridden regions have the lowest standard conditions of living and working (Trifiletti et al., 2017). The analysis of the present study is unique and adds scholarship to existing literature on terrorism stressors and job stressors. Overall, the data provide excellent support for the empirical investigation of terrorism and work stressors.

This research highlights the importance of effort-reward imbalance and organizational justice in risky areas. The success of staff working in these areas is a complex phenomenon, and although has not been extensively researched to date, there is still room to explain these phenomena in detail. This study is an early attempt to analyze the relationship between the ERI framework, sensitivity to terrorism, and emotional exhaustion, depersonalization, and lack of personal accomplishment at work separately. The study's findings will lead to future research on this significant issue faced by the world and conceptualize sensitivity to terrorism individually, enabling us to see its adverse effects and mitigate them in organizations operating in high risk areas.

## Data Availability Statement

The datasets presented in this article are not readily available because the data is highly confidential and cannot be shared as it may create a threat to the concerned workers. Requests to access the datasets should be directed to shuaib.ahmed@iba-suk.edu.pk.

## Ethics Statement

The studies involving human participants were reviewed and approved by University ethics committee. The patients/participants provided their written informed consent to participate in this study.

## Author Contributions

SASo, XHX, and AAG contributed to the design and implementation of the research, to the analysis of the results, and to the writing of the manuscript. SASh significantly helped us address the reviewer's comments in the manuscript. The author has made substantial contribution in data collection and revision of manuscript. He contributed in drafting the paper and also in interpretation of analysis part. All authors contributed to the article and approved the submitted version.

## Conflict of Interest

The authors declare that the research was conducted in the absence of any commercial or financial relationships that could be construed as a potential conflict of interest.
